# Combination Chemotherapy with Cisplatin and Chloroquine: Effect of Encapsulation in Micelles Formed by Self-Assembling Hybrid Dendritic–Linear–Dendritic Block Copolymers

**DOI:** 10.3390/ijms22105223

**Published:** 2021-05-14

**Authors:** Rebeca González-Pastor, Alexandre Lancelot, Violeta Morcuende-Ventura, María San Anselmo, Teresa Sierra, José L. Serrano, Pilar Martin-Duque

**Affiliations:** 1 Grupo de Terapia Génica y Celular, Instituto Aragonés de Ciencias de la Salud (IACS/IIS-Aragon), 50009 Zaragoza, Spain; glez.re@gmail.com; 2 Departamento de Química Organica, Instituto de Nanociencia y Materiales de Aragón (INMA), CSIC—University of Zaragoza, 50009 Zaragoza, Spain; alexandrelancelot@gmail.com (A.L.); violeta.m.v@csic.es (V.M.-V.); msananselmo@unizar.es (M.S.A.); tsierra@ctq.csic.es (T.S.); 3 Fundación Araid, 50018 Zaragoza, Spain; 4 Network of Biomedical Research Centers for Bioengineering, Biomaterials and Nanomedicine, CIBER-BBN, 28029 Madrid, Spain; 5 Universidad San Jorge, Villanueva de Gállego, 50830 Zaragoza, Spain

**Keywords:** chloroquine, cisplatin, combination chemotherapy, encapsulation, hybrid dendritic–linear–dendritic block copolymers

## Abstract

Clinical outcomes of conventional drug combinations are not ideal due to high toxicity to healthy tissues. Cisplatin (CDDP) is the standard component for many cancer treatments, yet its principal dose-limiting side effect is nephrotoxicity. Thus, CDDP is commonly used in combination with other drugs, such as the autophagy inhibitor chloroquine (CQ), to enhance tumor cell killing efficacy and prevent the development of chemoresistance. In addition, nanocarrier-based drug delivery systems can overcome chemotherapy limitations, decreasing side effects and increasing tumor accumulation. The aim of this study was to evaluate the toxicity of CQ and CDDP against tumor and non-tumor cells when used in a combined treatment. For this purpose, two types of micelles based on Pluronic^®^ F127 hybrid dendritic–linear–dendritic block copolymers (HDLDBCs) modified with polyester or poly(esteramide) dendrons derived from 2,2′-bis(hydroxymethyl)propionic acid (HDLDBC-bMPA) or 2,2′-bis(glycyloxymethyl)propionic acid (HDLDBC-bGMPA) were explored as delivery nanocarriers. Our results indicated that the combined treatment with HDLDBC-bMPA(CQ) or HDLDBC-bGMPA(CQ) and CDDP increased cytotoxicity in tumor cells compared to the single treatment with CDDP. Encapsulations demonstrated less short-term cytotoxicity individually or when used in combination compared to the free drugs. However, and more importantly, a low degree of cytotoxicity against non-tumor cells was maintained, even when drugs were given simultaneously.

## 1. Introduction

Combination chemotherapy has emerged as a more superior approach for cancer treatment than single-drug therapy, being able to achieve additive, potentiation or synergistic therapeutic effect and to overcome drug resistance [1,2]. While the use of a single drug could activate alternative molecular and cellular pathways in tumor cells, stimulating the appearance of chemoresistance mutations and tumor relapse, co-administration of two or more drugs with different mechanisms of action increases tumor cell death while reducing the chance of mutations evolving to develop cancer drug resistance [3,4,5,6]. A potential limitation to the efficacy of combination chemotherapy is associated to accumulating toxicities, and so it is often necessary to work at low concentrations of both drugs to avoid undesired side-effects [7,8]. Currently, different combinations of chemotherapeutic agents are successfully applied to treat ovarian, gastric, advanced colorectal, metastatic breast or non-small cell lung cancer [9,10,11,12,13]. In particular, cisplatin (CDDP) is a widely used chemotherapeutic agent known to cause DNA damage with a huge impact in the treatment of different types of cancer [14]. To limit its toxicity and increase its efficacy, it has been used in combination with bortezomib [15], ammonium chloride [16] or inhibitors of autophagy such as chloroquine (CQ) [17,18,19,20]. CQ is a known antimalarial that has been shown to induce cell death in a wide variety of tumor lines [21,22,23,24]. Besides, low doses of CQ produce a normalization of the tumor vasculature, improving perfusion and the efficacy of the treatment [25].

As new drug combinations and regimens of administration are being tested, it is important to carefully consider the biodistribution and pharmacokinetic profile of each drug in order to optimize the treatment [26]. In this context, drugs loaded into nanoparticle systems with sizes between 10–200 nm have been shown to have better pharmacokinetic profiles than drugs in their free form, as these nanosystems can passively or actively accumulate in tumors via the enhanced permeation and retention (EPR) effect [27]. Moreover, drug encapsulation limits systemic toxicity, which allows one to increase the doses administered [28,29]. Nanoparticle formulations based on polymers, liposomes, dendrimers or hydrogels have been developed to improve the solubility and increase the stability of chemotherapeutic drugs [30,31,32]. Additionally, their composition can be modulated to respond to microenvironment or external stimuli to precisely control the release rate of the chemotherapeutic agents, and their functionalization with targeting ligands favors binding to specific target cells [33,34]. As a result, these nanocarriers show greater blood circulation times and increased accumulation in the tumor [27,35]. Furthermore, nanocarriers allow the co-delivery of multiple therapeutics to the same site, limiting the variability in the biodistribution and dosage of each drug compared with the administration of two separate treatments [36,37,38,39,40].

**Figure 1:**
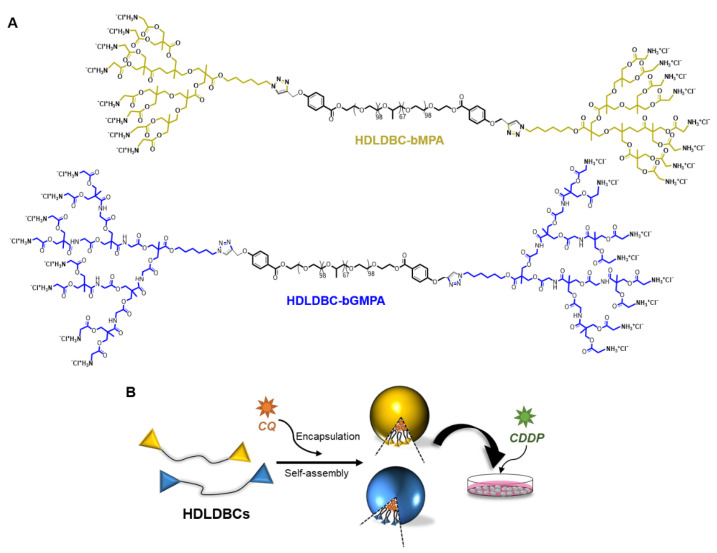
Representation of (**A**) chemical structures of the hybrid dendritic–linear–dendritic block copolymers (HDLDBCs) based on Pluronic^®^ F-127 and dendrons derived from 2,2′-bis(hydroxymethyl)propionic acid (bis-MPA) functionalized with glycine groups or 2,2′-bis(glycyloxymethyl)propionic acid (bis-GMPA). (**B**) Illustration of the structures obtained in the encapsulation of chloroquine (CQ) in the HDLDBC-bMPA (yellow) or HDLDBC-bGMPA (blue) for their use in vitro in combination with cisplatin (CDDP).

Based on these considerations and the excellent results achieved with Pluronic^®^ F-127 and chemotherapeutics in previous studies [41,42], in this work, we used two dendritic–linear–dendritic hybrid block copolymers (HDLDBCs) based on Pluronic^®^ F-127, which was functionalized at both termini with bis-MPA (2,2′-bis(hydromethyl)propionic acid) (HDLDBC-bMPA) or bis-GMPA (2,2′-bis(glycyloxymethyl)propionic acid) dendrons (HDLDBC-bGMPA) as drug encapsulation carriers for combination chemotherapy with CDDP and CQ (Figure 1). The HDLDBCs (described for the first time by Frechet et al. [43]) are composed of a linear polymer with dendritic fragments in both extremities and, therefore, combine characteristics derived from the rigid globular and branched parts of the ends with the high mobility of the central linear polymer chain [44]. In these amphiphilic block copolymers, the rigidity of the dendritic ends favors the stabilization of supramolecular structures [45] and it is possible to increase the capacity of encapsulation, since the drug is not only enclosed in the dendritic cavities, but also occupies the spaces between molecules [46]. Both bis-MPA and bis-GMPA HDLDBCs have already been used in previous studies of our group for the encapsulation of CQ [47,48], and camptothecin [49]. Here, we show promising results in terms of the reduction in side-effects of the combination treatment with CQ encapsulated in HDLDBC-bMPA or HDLDBC-bGMPA and free CDDP compared to the treatment with free CQ and free CDDP, based on the cytotoxicity against tumor cells and high degree of biocompatibility towards non-tumor cells.

## 2. Results

### 2.1. Synthesis and Design of the Hybrid Dendritic–Linear–Dendritic Block Copolymers (HDLDBCs)

In this study, two amphiphilic hybrid dendritic–linear–dendritic block copolymers (HDLDBCs) (Figure 1) have been synthesized following previously published protocols [47,48]. Both derivatives were based on a central commercial amphiphilic linear triblock copolymer Pluronic^®^ F-127 (PEO99-PPO67-PEO99), which was functionalized at its two terminal positions by third generation amino-terminated bis-MPA dendrons or by third generation bis-GMPA dendrons, named HDLDC-bMPA and HDLDBC-bGMPA, respectively. The union between linear polymer and dendrons was carried out by copper (I) azide–alkyne cycloaddition click-chemistry reaction. HDLDBC-bMPA and HDLDBC-bGMPA have an average molecular weight of 16.3 and 17.0 kg·mol^−1^, respectively, and both showed high biocompatibility in a variety of different cells lines [47,48].

### 2.2. Self-Assembly of the HDLDBCs

**Figure 2:**
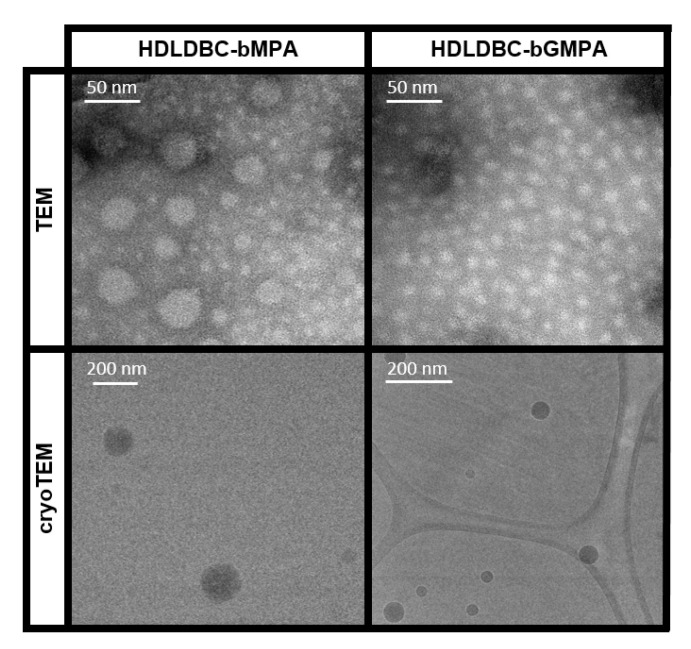
Characterization of the nanocarriers. TEM and cryoTEM images of HDLDBC-bMPA and HDLDBC-bGMPA.

Both HDLDBCs can self-assemble in water. The critical micellar concentration (CMC) of HDLDBC-bGMPA was previously determined using the Nile Red technique, being of 6 × 10^−5^ mol·L^−1^ [48]. Following the same procedure, the CMC of HDLDBC-bMPA was determined for this study, being of 3 × 10^−5^ mol·L^−1^. Both values were slightly slower than the CMC determined for Pluronic^®^ F-127 using the same technique, namely 1 × 10^−4^ mol·L^−1^. The two dendrons, located at the extremities of Pluronic^®^ F-127, may favor the stabilization of the micelles at lower concentration, increasing molecular interactions. Additionally, the size and morphology of the nanocarriers was studied by TEM and cryoTEM microscopy, as depicted in Figure 2 and Table 1. Both HDLDBCs produced rounded micelles. Two size-populations for HDLDBC-bMPA were observed in TEM images: one population with an average diameter of 13 ± 3 nm and another population, less represented, with an average diameter of 22 ± 4 nm. Only one size population was observed for HDLDBC-bGMPA with an average diameter of 13 ± 3 nm. CryoTEM, which allows for the observation of the samples as they are in aqueous solution [50], afforded images in which two size-populations were observed for HDLDBC-bMPA, i.e., small micelles with diameters around 60 nm and bigger aggregates with sizes near 150 nm. In the case of HDLDBC-bGMPA, only one size-population was observed with diameters in the range of 40 to 70 nm.

DLS measurements were also conducted to study the hydrodynamic size of the aggregates. Table 1 gathers intensity average DLS values, together with TEM and cryoTEM size measurements. DLS also detected two size-populations for HDLDBC-bMPA and one size-population for HDLDBC-bGMPA.

### 2.3. Cell Internalization of HDLDBC Micelles

**Figure 3:**
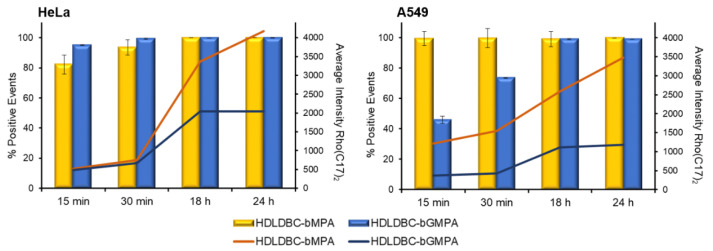
Internalization curves of HDLDBCs (Rho(C17)_2_) in HeLa and A549 cells. Flow cytometry analysis after incubation of cells with rhodamine-labeled HDLDBC-bMPA or HDLDBC-bGMPA at different time points. Yellow and blue bars represent the percentage of rhodamine-positive events relative to the total number of events recorded. Orange and blue lines represent the average rhodamine intensity of each population at different time-points.

In order to study cellular uptake of the nanocarriers by flow cytometry, a lipophilic modified rhodamine B (Rho(C17)_2_) was encapsulated in HDLDBC-bMPA and HDLDBC-bGMPA. Encapsulation of this low-water soluble rhodamine has already been described to be useful for tracking nanocarrier internalization inside cells [48,49]. Although, in general, the encapsulations were quickly internalized in tumor cells, HeLa and A549 cells, and non-tumor cells, fibroblasts (Fdh), with at least 90% of the population being rhodamine-positive after 30 min of incubation, HDLDBC-bGMPA(Rho(C17)_2_) nanoaggregates were internalized somewhat slower in A549 cells, reaching 73% of target cells at early time points (Figure 3 and Figure S1). The average intensity of rhodamine that correlates with the amount of encapsulations internalized greatly increases up to 18 h of incubation, reaching a plateau around 24 h. Additionally, results showed a reduced cellular uptake of the HDLDBC-bGMPA(Rho(C17)_2_) nanoaggregates compared to the HDLDBC-bMPA(Rho(C17)_2_) nanoaggregates. Of note, as previously described, no release of Rho(C17)_2_ from HDLDBC-bGMPA(Rho(C17)_2_) nanoaggregates was observed in 72 h experiments at 37 °C [48], and less than 2% of Rho(C17)_2_ was released from the encapsulations after 24 h of incubation (Figure S2). These experiments ensure that the signal detected inside the cells belongs to the nanoaggregates and not to the free Rho(C17)_2_.

### 2.4. Drug Encapsulation in HDLDBC Nanoaggregates and Releasing Profiles

While the oil-in-water procedure allowed us to individually encapsulate CQ and CDDP in the HDLDBCs, the efficiency of encapsulation (EE) was different for the two drugs, being a much more efficient and reproducible process for the encapsulation of CQ compared to CDDP (Table 2). Indeed, the amount of CQ encapsulated within any of the HDLDBCs exceeded by more than ten times that of CDDP.

According to DLS measurements, all aggregates presented two size-populations after drug encapsulation (Table 2 and Figure S3 for TEM images). Their hydrodynamic diameter also increased, with the increase being more pronounced for HDLDBC-bGMPA than for HDLDBC-bMPA. Surprisingly and despite its lower encapsulation efficiency, CDDP encapsulation triggered the formation of bigger aggregates than CQ.

We next investigated the in vitro release of CQ from HDLDBC-bMPA in PBS (Figure S4). In the initial stage, there is a burst release at 37 °C, where 23% of CQ is released in 2 h and another 26% is gradually released within 20 h. From this time on, CQ remained encapsulated for at least 4 more days. Similar releasing profiles were described for CQ encapsulated in HDLDBC-bGMPA [48].

### 2.5. Effect of Free and Encapsulated CQ and CDDP on the Proliferation of HeLa and A549 Cells

**Figure 4:**
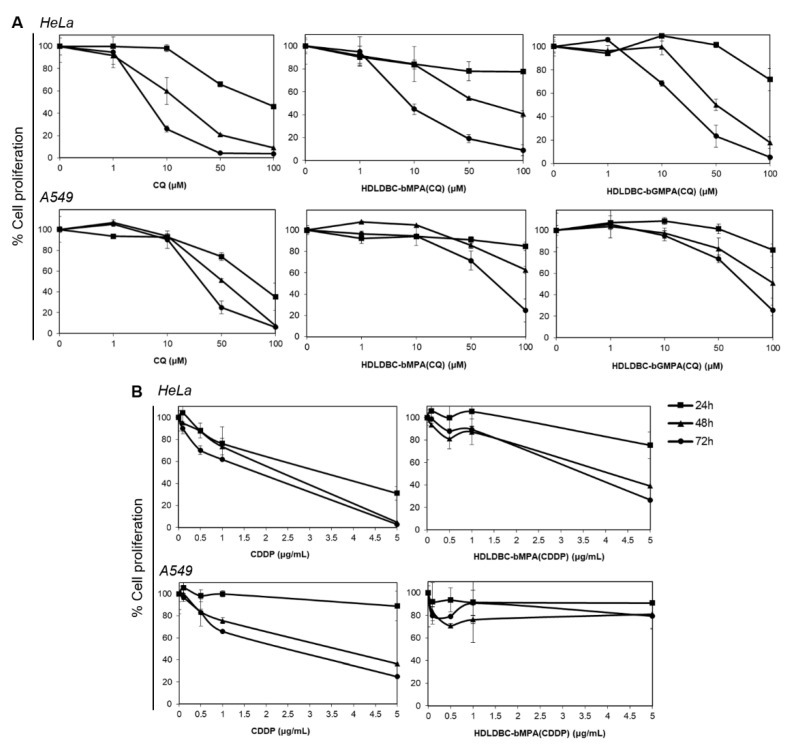
Effect of (**A**) CQ and (**B**) CDDP free or encapsulated on HDLDBC-bMPA or HDLDBC-bGMPA on the proliferative activity of Hela cells and A549 cells at 24 h, 48 h and 72 h determined by the AlamarBlue assay. The y-axis represents the proliferation rate, calculated as the ratio to control untreated cells at each time point. The CQ and CDDP-induced inhibitory effect was time and dose-dependent. Values are given as mean ± SD (*n* = 3).

Cell proliferation curves were generated in tumor cell lines HeLa and A549 after incubation with CQ and CDDP free and encapsulated in HDLDBC-bMPA or HDLDBC-bGMPA. No significant differences were found between 18 h and 24 h of incubation; therefore, only 24–72 h data are shown. Encapsulation of CQ and CDDP resulted in decreased cytotoxicity towards both cells lines compared to the free drugs (Figure 4).

CQ at doses lower than 1 µM had no inhibitory effect on HeLa or A549 cells, either in free form or encapsulated, and inhibited proliferation in a time- and dose-dependent manner at higher doses (Figure 4A). Whereas free CQ exhibited cytotoxicity in HeLa cells at concentrations over 50 µM after 24 h of incubation and above 1 µM in a 48–72 h incubation period, HDLDBC-bMPA(CQ) and HDLDBC-bGMPA(CQ) had minimal inhibitory effect at 24 h and were cytotoxic at concentrations over 10 μM in a 48 h and 72 h treatment. Although growth inhibition in A549 cells followed a similar pattern, CC50 values were higher (Table 3), with cell proliferation decreasing after 48–72 h treatment with 10 µM of free CQ or 50 µM of HDLDBC-bMPA(CQ) or HDLDBC-bGMPA(CQ).

CDDP also the inhibited growth of HeLa and A549 cells in a dose- and time-dependent manner (Figure 4B). Cell growth levels remained above 70% after incubation with both free CDDP and CDDP encapsulated in HDLDBC-bMPA at concentrations of 1 μg/mL, while in HeLa cells, concentrations of 5 μg/mL of CDDP decreased cell proliferation below 30% after 24 h, HDLDBC-bMPA(CDDP) inhibited cell growth to 40% after 48 h. In contrast, HDLDBC-bMPA(CDDP) had minimal inhibitory effect on A549 cell proliferation, exhibiting values over 75% even after 72 h of incubation. Thus, CC50 values for HDLDBC-bMPA(CDDP) were two to three-fold higher in Hela cells and at least two-fold higher in A549 cells compared to the values obtained with free CDDP (Table 3).

These results indicate that encapsulation of CQ and CDDP in HDLDBC-bMPA or HDLDBC-bGMPA decreased cytotoxicity against HeLa and A549 cells, and thus the CC50 values were higher compared to the values of the free drugs. Based on these results, a range of concentrations of CQ (40 and 80 μM) and CDDP (0, 0.5, 1 and 3 μg/mL) were selected for the combination study.

**Figure 5:**
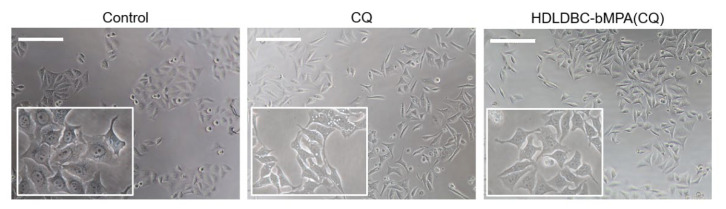
Effect of CQ on HeLa cell morphology. Cells were treated with 40 µM of CQ or encapsulated HDLDBC-bMPA(CQ) for 24 h. Cell damage was evaluated by analysis of cell micrographs obtained on a phase contrast microscope. Scale = 200 µm (4×) and inset (20×).

Image analysis of micrographs revealed changes in cell morphology after treatment of Hela cells with CQ to adopt a more elongated shape, together with the presence of heavily vacuolated cells. This phenomenon was more evident for the treatment with free CQ compared to HDLDBC-bMPA(CQ) (Figure 5).

### 2.6. Effect of Combination of CQ and CDDP on the Proliferation of Tumor Cells and Fibroblasts

Based on the results obtained during drug encapsulation processes and observed in cell proliferation curves (Section 2.3 and Section 2.5), we decided to focus our investigation on a combined chemotherapy protocol constituted of a pre-treatment with encapsulated CQ for 18 h followed by a free CDDP treatment for 48 h and compared its efficacy against tumor and non-tumor cell lines. This strategy allowed us to exploit the good and reproducible encapsulation efficiency of CQ within the HDLDBC micelles and to control drug activity in both tumor cell lines. Encapsulated CDDP was not further studied due to its low encapsulation efficiency within the HDLDBC micelles and its low drug activity against tumor cells (Figure S5). Moreover, the simultaneous administration of both drugs encapsulated separately or the administration of the double encapsulation of CQ and CDDP within HDLDBC-bMPA did not induce significant cytotoxicity against tumor cells, maintaining cell viability between 85–100% after 72 h of incubation (see SI for double encapsulation protocol and Figure S5).

**Figure 6:**
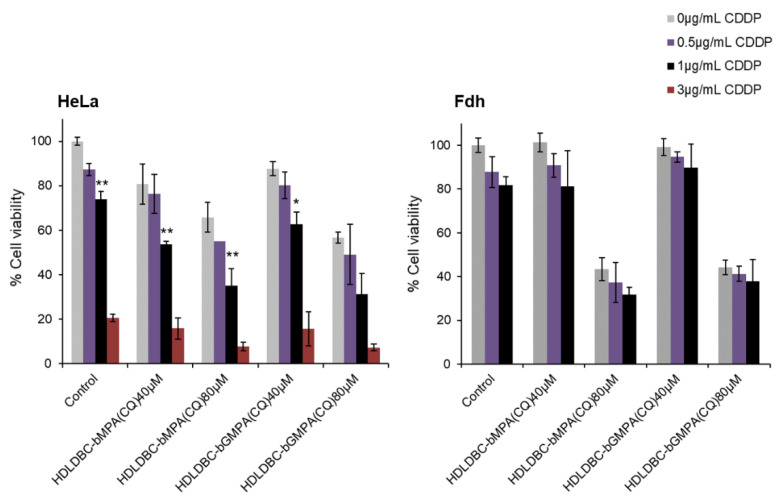
Effect of pre-treatment with CQ encapsulated in HDLDBC-bMPA or HDLDBC-bGMPA and treatment with free CDDP. Cell viability of HeLa cells and human fibroblasts (Fdh) after pre-treatment with 40 μM or 80 μM of encapsulated CQ during 18 h and additional treatment with different concentrations of free CDDP during 48 h. Values are given as mean ± SD (** *p* < 0.01 and * *p* < 0.05, compared to encapsulated CQ combined with 0 μg/mL of free CDDP).

Hence, in order to select the concentration of each drug to be used, we considered the doses that produced a mild inhibition of cell proliferation separately and analyzed cell viability against HeLa cells and fibroblasts (Fdh) to achieve the best combined effect (Figure 6).

Pre-treatment of cells with encapsulated CQ for 18 h prior to exposure to free CDDP enhanced the inhibitory effect. The cell viability of Hela cells decreased between 10–20% compared to the control when treated with 40 µM of CQ and between 30–40% when treated with 80 µM of CQ, depending on the concentration of free CDDP used. While the cell viability of Fdh was maintained above 80% and no significant differences were found when incubating with 40 µM of encapsulated CQ and/or 0.5–1 μg/mL of free CDDP, a 60% decrease in cell viability was reported when pre-treating with 80 µM of encapsulated CQ. No significant differences were seen in terms of cell viability between the encapsulation of CQ in HDLDBC-bMPA and HDLDBC-bGMPA. As the objective was to use a combination treatment that inhibits the growth of HeLa cells and at the same time marginally affects cell viability of the non-tumor cells Fdh, a concentration of 40 μM of encapsulated CQ and a concentration of 1 μg/mL of CDDP were selected for the following experiments, which decreased the viability of HeLa cells to 50% of the control, in contrast to 85% of cell viability seen in Fdh.

### 2.7. Effect on Cell Proliferation of a Combined Treatment with CQ Encapsulated in HDLDBCs and Free CDDP Compared to the Use of Free Drugs and Degree of Interaction of the Drugs

**Figure 7:**
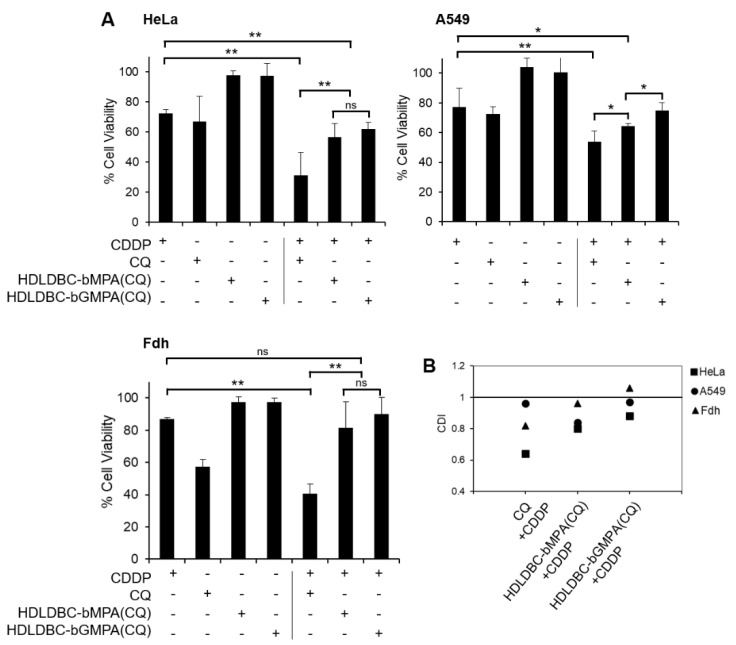
Effect of pre-treatment with 40µM of free CQ, HDLDBC-bMPA(CQ) or HDLDBC-bGMPA(CQ) in the treatment with free CDDP in HeLa and A549 cells and fibroblasts (Fdh). (**A**) Cell viability after single treatment with 40 µM of CQ or 1 µg/mL of CDDP (left side) or combined treatment (right side) (** *p* < 0.01 and * *p* < 0.05; ns: not significant). (**B**) The CDI value represents a measure of the degree of interaction between two drugs. When this value is between 0.7 and 1, it indicates a low–moderate synergism; when CDI is between 0.3 and 0.7, it indicates a high synergism.

Once we found a combined treatment with encapsulated CQ and free CDDP that significantly affected the viability of tumor cells while marginally affecting non-tumor cells, we compared this treatment with a treatment using free CQ. Hence, cells were pre-treated for 18 h with 40 µM of free CQ or CQ encapsulated in HDLDBC-bMPA or HDLDBC-bGMPA. Subsequently, cells were treated with 1 µg/mL of CDDP for 48 h. The results are shown in Figure 7.

Single treatment with free CDDP or free CQ reduced cell viability of HeLa and A549 cells to approximately 70% and 75% of the control, respectively. On the other hand, treatment with encapsulated CQ had no effect on cell viability. Although both free and encapsulated CQ enhanced the inhibitory effect of CDDP, pre-treatment with free CQ had a greater effect, reducing viability to 31% in HeLa cells and 54% in A549 cells, in contrast to the reduction in cell viability to around 60% in HeLa cells and around 70% in A549 cells when pre-treating with HDLDBC-bMPA(CQ) and HDLDBC-bGMPA(CQ). Even though the results showed no significant differences in cell viability in Hela cells between the encapsulations, overall, HDLDBC-bMPA(CQ) was more efficient when used in combination with CDDP. Importantly, while free CQ in combination with free CDDP presented great cytotoxicity against Fdh and decreased cell viability to 40% of the control, cell viability remained at 81% and 89% when Fdh were treated with HDLDBC-bMPA(CQ) or HDLDBC-bGMPA(CQ) combined with CDDP. The degree of interaction between CQ and CDDP indicated a high degree of synergism against HeLa cells and a low degree of synergism against A549 cells when pre-treating cells with free CQ (Figure 7B). On the other hand, there was only moderate to low synergism between encapsulated CQ and free CDDP against both cell lines.

**Figure 8:**
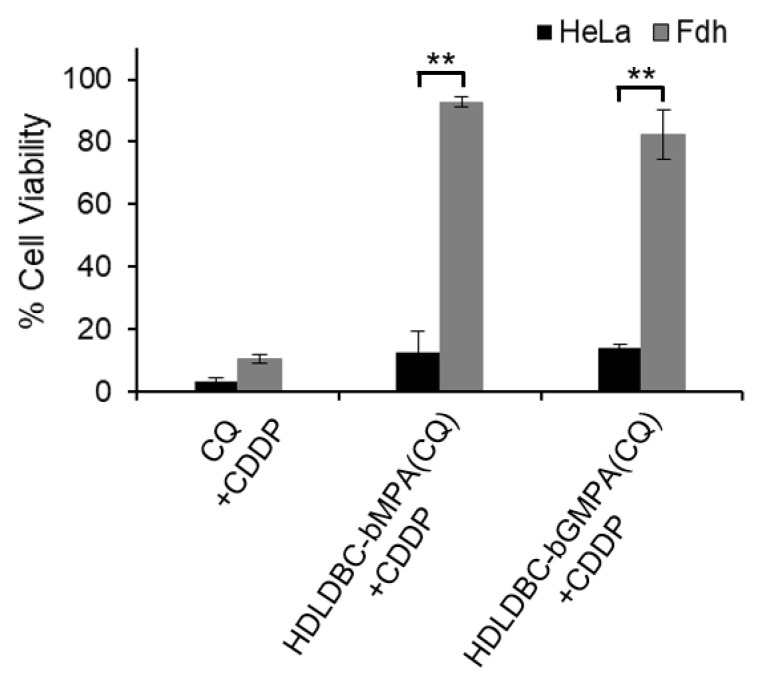
Cell viability after the simultaneous treatment with CQ and CDDP on HeLa and Fdh. Cells were incubated with 40 µM of free CQ or CQ encapsulated in HDLDBC-bMPA and 1 µg/mL of free CDDP during 72 h (** *p* < 0.01).

In addition, a striking difference in terms of cell toxicity between free and encapsulated CQ was observed when both drugs were simultaneously incubated for 72 h with HeLa cells and Fdh. While treating cells with free CQ and free CDDP reduced cell viability of HeLa cells and Fdh below 10%, treating cells with HDLDBC-bMPA(CQ) or HDLDBC-bGMPA(CQ) and CDDP similarly reduced cell viability of HeLa cells to 12%, but only reduced cell viability of Fdh to 93% and 82%, respectively (Figure 8).

These results indicated that the combined treatment with HDLDBC-bMPA(CQ) or HDLDBC-bGMPA(CQ) and CDDP increased cytotoxicity in tumor cells compared to the single treatment with CDDP while maintaining a high degree of biocompatibility against non-tumor cells.

### 2.8. Cell Cycle Changes and Induction of Apoptosis after Combination Treatment with Encapsulated CQ and Free CDDP

**Figure 9:**
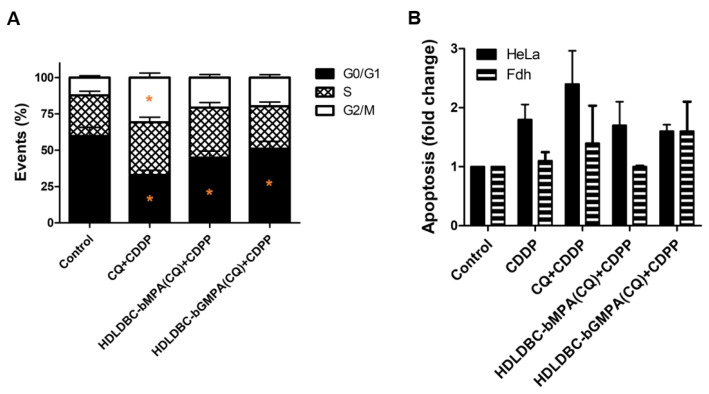
Cell cycle changes and induction of apoptosis was analyzed by flow cytometry after treatment with 40 µM of free CQ or CQ encapsulated in HDLDBC-bMPA for 18 h and/or 1 µg/mL of CDDP for an additional 48 h. (**A**) HeLa cells were stained with propidium iodide (PI) and cell cycle distribution was measured after 72 h of treatment (* indicates alterations > 10% compared to control group). (**B**) HeLa cells and Fdh were double-stained with Annexin V-FITC/PI to detect the contribution of apoptosis and necrosis after treatment for 72 h. Apoptotic populations are presented as fold change of control group.

Pre-treatment with CQ for 18 h followed by treatment with CDDP for 48 h significantly arrested the cell cycle of HeLa cells at G2/M and slightly at S phase compared to the control (Figure 9A and Figure S6A); this modulation was greater with free CQ compared to encapsulated CQ (percentage of cells in G2/M: 31% vs. 21% vs. 20% for free CQ vs. HDLDBC-bMPA(CQ) vs. HDLDBC-bGMPA(CQ), respectively).

Even though the percentage of apoptotic cells was slightly increased by pre-treatment with free CQ combined with CDDP compared to treatment with CDDP alone (0.6-fold increase), the pre-treatment with HDLDBC-bMPA(CQ) or HDLDBC-bGMPA(CQ) did not lead to an increase in apoptotic cells over the treatment with CDDP alone (Figure 9B and Figure S6B). No significant differences were seen in Fdh after the combination treatment.

These results confirm that, although cell cycle is blocked in G2/M when cells are pre-treated with HDLDBC-bMPA(CQ) or HDLDBC-bGMPA(CQ) followed by treatment with CDDP, these changes are not enough to significantly increase the apoptotic effect of CDDP.

## 3. Discussion

Cisplatin (CDDP) is one of the most used anticancer drugs for the treatment of prostate, ovarian, head and neck, bladder and lung cancer. CDDP interacts with DNA and forms DNA adducts that result in the interference with DNA synthesis and repair, thus inducing cell apoptosis [51]. However, the clinical effectiveness of CDDP is usually limited by side toxicity, particularly nephrotoxicity, and drug resistance [52,53,54], largely by means of induction of autophagy [55] and faster repairing of DNA lesions [56,57,58,59,60]. In order to increase the therapeutic efficacy and reduce the side effects, CDDP is commonly used in combination with other drugs [51]. In this context, chloroquine (CQ), which has long been used as an anti-malarial and anti-rheumatic drug, sensitizes cancer cells to chemotherapeutic drugs through the prevention of autophagy [61,62,63,64]. As both CDDP and CQ are nonselective and can damage healthy tissues, reducing their effective doses and increasing delivery into tumor cells is key and can be accomplished by the encapsulation of the drugs in nanoparticles [30,65]. These nanostructures protect the drugs from degradation and can assist in overcoming or avoiding chemoresistance while minimizing systemic toxicity. CDDP [66,67,68] and CQ [47,48,69] have already been successfully formulated into different types of nanocarriers.

Based on our previous knowledge on CQ encapsulation into micelle carriers [47,48], our study focused on the combination treatment with CQ and CDDP loaded into micelles formed by hybrid dendritic–linear–dendritic block copolymers (HDLDBCs) that bear polyester or poly(esteramide) dendrons in the extremities.

Here, we confirmed that CQ was progressively released from HDLDBC-bMPA in PBS at 37 °C during 24 h; after this time point, CQ remained encapsulated for the rest of the experiment. A similar release profile was already shown when CQ was encapsulated in HDLDBC-bGMPA, and it was then discussed that the polymer architecture might prevent an adequate release of the drug and thus compromise the efficacy of the treatment [48]. The internalization of HDLDBC-bMPA(Rho(C17)_2_) and HDLDBC-bGMPA(Rho(C17)_2_) nanoaggregates showed 90% of rhodamine-positive population as early as 30 min after incubation in HeLa and A549 cells, following progressive uptake and accumulation of the encapsulations for a period of 18 h, as shown by the increase in the average intensity signal of rhodamine. On the other hand, it seems that HDLDBC-bGMPA(Rho(C17)_2_) nanoaggregates were internalized to a lesser extent in both cell lines, and this reduced uptake was more evident in A549 cells. Previous studies have described HDLDBC-bGMPA nanoaggregates to be mainly distributed in the cytoplasm of non-tumor cells after internalization [48]. Based on the size and spherical morphology of the micelles, HDLDBCs nanoaggregates could be internalized by clathrin-mediated and caveolae-mediated endocytosis [70].

The evaluation of the cytotoxicity of free CQ and encapsulated CQ showed inhibition of HeLa and A549 cell proliferation in a time- and dose-dependent manner at doses higher than 1 µM. In all cases, a more than two-fold increase in the concentration of the encapsulated CQ is required to produce the same levels of growth inhibition as free CQ. The CC50 values of free CQ in HeLa cells ranged from 16 μM at 48 h to 8 μM at 72 h compared to CC50 values of HDLDBC-bMPA(CQ), which ranged from 58 μM at 48 h to 20 μM at 72 h. CC50 values of HDLDBC-bGMPA(CQ) were higher than those reported at 72 h for HDLDBC-bMPA(CQ), probably related to the reduced cell uptake. On the other hand, the CC50 values of free and encapsulated CQ in A549 cells were higher than CC50 values in HeLa cells. The CC50 values obtained for HDLDBC-bMPA(CQ) and HDLDBC-bGMPA(CQ) are higher than those reported for other formulations of CQ [71,72,73].

Differences in cell proliferation inhibition of free CQ compared to encapsulated CQ are likely related to the gradual release of the drug, where 50% of CQ was released from HDLDBC-bMPA and HDLDBC-bGMPA after 24 h, while the nanoaggregates were being internalized. This was also evident by the presence of less vacuoles when cells were treated with HDLDBC-bGMPA(CQ). Once inside the cells, CQ becomes available more rapidly as HDLDBCs undergo hydrolytic and enzymatic degradation, as already established for block copolymers based on Pluronic^®^ F-127 [74,75,76] and bis-MPA and bis-GMPA dendrimers [77,78]. Nanocarriers based on similar structural components can take more than a week to degrade, depending on temperature and solution conditions, which will explain the absence of the complete release of CQ from HDLDBC-bMPA and HDLDBC-bGMPA and the higher CC50 values compared to CC50 values of free CQ. Thus, incubation for a longer period may improve cytotoxic efficacy. Nonetheless, the slow release could be beneficial in vivo, allowing for a second burst of CQ release once inside the tumor. Overall, these results indicated that encapsulated CQ with free CDDP could inhibit proliferation like free CQ with free CDDP, though in an extended process due to its sustained released from HDLDBC micelles.

Similar to the inhibitory effect of CQ, clinically relevant concentrations of free CDDP [79] showed concentration and time-dependent reduction in cell proliferation in both cell lines, with A549 cells being more resistant compared to HeLa cells. Conversely, while HDLDBC-bMPA(CDDP) inhibited the growth of HeLa cells in a dose- and time-dependent manner and CC50 values were two to three-fold higher compared to CC50 values for free CDDP, A549 cell proliferation remained unchanged when cells where incubated with the encapsulated CDDP up to a concentration of 5 μg/mL. Different studies reported reduced cytotoxicity in vitro of micelle-encapsulated CDDP compared to free CDDP based on the release prolife, although in vivo results demonstrated more tumor accumulation and higher efficacy [80,81,82].

Several clinical trials have reported that sequential chemotherapy could maintain antitumor efficacy while reducing the incidence of toxicity [83,84] compared to simultaneous chemotherapy. Based on this, on the sensitizing ability of CQ [85] and our results, we decided to pre-treat the cells with encapsulated CQ for 18 h and then treat the cells with free CDDP for an additional 48 h. The selection of the final doses to be used was made based on the treatment that inhibited the growth of tumor cells and at the same time minimally affected cell viability of non-tumor cells (Figure 6). Pre-treatment with lower concentrations of encapsulated CQ (40 μM) resulted in higher cell growth inhibition compared to treatment with CDDP alone. Of note, the increase in cytotoxicity against Fdh obtained with CQ at higher doses (80 μM) was significant, independent of the concentration of CDDP. As expected, co-treatment of tumor cells with CQ and CDDP significantly enhanced the inhibitory effect compared to CQ or CDDP treatment alone, either free or encapsulated, though there was only moderate synergism between HDLDBC-bMPA(CQ) and free CDDP. These results showed that A549 cells were less sensitive to the combined treatment with free and encapsulated CQ compared to HeLa cells. More importantly, although Fdh were similarly affected by the combined treatment with free CQ and free CDDP as HeLa cells, combinatorial treatment of Fdh with HDLDBC-bMPA(CQ) or HDLDBC-bGMPA(CQ) and free CDDP maintained cell viability between 80–90%, respectively.

The progression of the cell cycle was analyzed 72 h after sequential treatment with CQ, HDLDBC-bMPA(CQ) or HDLDBC-bGMPA(CQ) and free CDDP. Treatment arrested cancer cells at the G2/M phase, although the impact on apoptosis was not as notable. An increase in CQ concentration would potentiate DNA damage and so apoptosis [86] and the effect of CDDP [87] but would also increase toxicity against Fdh.

The double encapsulation of CQ and CDDP into HDLDBC-bMPA did not work as expected (Figure S4). One possible explanation is that due to the disposition of the drugs within the aggregates, they are inactivated. Interestingly, while simultaneous treatment with free drugs had a huge impact on both tumor and non-tumor cells, simultaneous treatment with HDLDBC-bMPA(CQ) or HDLDBC-bGMPA(CQ) and free CDDP resulted in high levels of cytotoxicity only against tumor cells. A possible explanation of this effect could be the reduced ability of Fdh to retain CQ encapsulations compared to tumor cells, as well as changes induced by cisplatin on membrane properties that reduce drug uptake and alter vesicular trafficking [62,88,89]. This opens the possibility of safely using the drugs simultaneously to achieve greater tumor cell killing without reducing the concentrations [85,90].

In conclusion, combination treatment with HDLDBC-bMPA(CQ) or HDLDBC-bGMPA(CQ) and free CDDP significantly increased cytotoxicity in tumor cells compared to the single treatment with CDDP while maintaining a high degree of biocompatibility towards non-tumor cells. Future work should optimize the encapsulation system to obtain a better control over the release of the drugs and a more efficient double encapsulation, adjusting loading ratios and spatial–temporal release for improved anti-tumor effect and the reduced development of drug-resistance. Additionally, in vivo testing should analyze biodistribution and long-term toxicity.

## 4. Materials and Methods

### 4.1. Reagents

The reactive and organic solvents were purchased from Sigma-Aldrich^®^ (Saint Louis, MO, USA), Acros^TM^ (Geel, Belgium) or Scharlab S.L (Sentmenat, Spain). The dialysis cassettes, Slide-A-Lyzer^TM^ (Thermo Fisher, Madrid, Spain) of cellulose acetate with 2000 Da cut-off were purchased from Thermo-Fisher Scientific (Waltham, MA, USA). In particular, chloroquine diphosphate salt (purity ≥98.5%) and cis-platin (purity: 99.99%) were purchased form Acros^TM^ (Geel, Belgium).

### 4.2. Characterization of the Hybrid Dendritic–Linear–Dendritic Block Copolymers (HDLDBCs)

The critical micellar concentration (CMC) was determined for Pluronic^®^ F-127 and the HDLDBC-bMPA using the Red Nile technique. An amount of 2 mL of distilled water solutions of the macromolecular derivative with concentrations ranging from 0.1 to 5 mg mL^−1^ were prepared at 25 °C. Nile Red was dissolved in ethanol at a concentration of 0.25 mM and 10 μL of this solution was added to each sample. The mixtures were then stirred at room temperature for 1 h in the dark with an orbital shaker. The fluorescence emission spectrum of each solution was recorded with a PerkinElmer (Waltham, USA) LS 55 fluorimeter after excitation at λex = 550 nm.

Transmission electron microscopy (TEM) images were obtained with a FEI TECNAI T20 (FEI^TM^, Hillsboro, USA) with beam power of 200 kV using holey carbon film 300 mesh coppered grids provided by Agar Scientific Ltd. (Stantsed, UK). A droplet of an aqueous solution of the samples at the concentration of 1 mg/mL at 25 °C was deposited on the grid and allowed to penetrate for 30 s; the excess of the aqueous solution was removed using a filter paper. A phosphotungstic acid, 3% aqueous solution was used as negative stain; a droplet of the stain solution was deposited on the sample grid and let penetrate during 10 s; the excess of the staining aqueous solution was removed using a filter paper. The grid was dried during at least 24 h under atmospheric pressure. The average size of the different aggregates measured by TEM was obtained by comparing at least 200 aggregates in at least 6 photos.

Cryogenic transmission electron microscopy (cryoTEM) images were obtained with a FEI TECNAI T20 (FEI^TM^, Hillsboro, USA) with beam power of 200 kV using quantifoil carbon film 300 mesh coppered grid or lacey carbon film 300 mesh coppered grids provided by Agar Scientific Ltd. (Stantsed, UK). Quantifoil grids were previously ionized using a plasma cleaner whereas lacey carbon grids were previously ionized using a glow discharge apparatus. A droplet (60 μL) of an aqueous solution of the samples was deposited on the grid. Sample vitrification was automatically processed using a vitrobot (FEI) and performed in liquid ethane. A specific sample holder, Gatan (Pleasanton, CA, USA) for cold samples, was used to stock the grids in liquid nitrogen prior to observation with the microscope.

Dynamic light scattering (DLS) measurements were performed with Malvern Instruments (Malvern, UK) Nano ZS and a Brookhaven Instruments Corporation (Holtsville, USA) 90 plus particle size analyzer at concentrations of 1 mg/mL at 25 °C.

### 4.3. Preparation of Encapsulation of CQ, CDDP and Rhodamine and Drug Release Assays

Chloroquine diphosphate salts (CQ) and cisplatin (CDDP) were encapsulated within the HDLDBCs following the oil-in-water procedure previously employed by us [47]. Briefly, the HDLDBC and the drugs were dissolved in a mixture of dichloromethane and distilled water (1:1) at a feeding ratio of (1:1) (wHDLDBC:wdrug) for CQ and at a feeding ratio of (1:0.25) (wHDLDBC:wdrug) for CDDP. The samples were vigorously stirred at room temperature employing an orbital shaker under ventilation until complete evaporation of DCM (around 2 h). The non-encapsulated drugs were removed by dialysis at 4 °C (regenerated cellulose membrane, Slide-A-Lyzer devices, MW 1000 Da cut-off, Thermo Scientific, (Waltham, USA)) against distilled water (200 mL) during 16 h. The amounts of encapsulated drugs were indirectly determined. The quantities of chloroquine present in the dialysis water were measured by UV–vis spectrometry (Varian Cary50 Probe UV–visible spectrophotometer, Agilent Technologies, Las Rozas, Spain) at the wavelength of λ_A_(CQ) = 345 nm. The quantities of CDDP present in the dialysis water were measured by inductively coupled plasma atomic emission spectrometry (ICP-AES, Servicio de Análisis Químico, SAI-Universidad de Zaragoza).

In order to study the internalization of the nanoaggregates formed by the HDLDBCs, they were labeled by a low-water soluble fluorophore derived from rhodamine B (Rho(C17)_2_). This fluorophore was first encapsulated within the HDLDBCs following the same procedure at a feeding ratio of (1:0.15) (wHDLDBC:wRho(C17)_2_). As it is insoluble in water and as no solid precipitate were detected after the encapsulation procedure, it was assumed that all the fluorophores could be encapsulated within the HDLDBC (final concentration of Rho(C17)_2_ = 150 μg/mL and HDLDBC final concentration 1 mg/mL).

CQ release was studied by dialysis. An amount of 2 mL of HDLDBC-bMPA(CQ) were dialyzed against distilled PBS (200 mL) using a regenerated cellulose membrane, Slide-A-Lyzer devices (Waltham, MA, USA), MW 1000 Da cut-off, Thermo Scientific. The quantity of CQ released in the PBS was measured by taking 2 mL aliquots from the PBS water dialysis at different times for 120 h.

### 4.4. Cell Lines and Cell Culture

HeLa and A549 were obtained from Cancer Research UK and human dermal fibroblast (Fdh) were obtained from ATCC and cultured in Dulbecco’s modified eagle medium (DMEM) GlutaMAXTM-1 supplemented with 10% fetal bovine serum (FBS) (Gibco, ThermoFisher Scientific) and 1% penicillin/streptomycin/amphotericin (Biowest). All cell lines were maintained at 37 °C in a humidified atmosphere at 5% CO_2_.

### 4.5. Encapsulations Internalization

Cells were seeded at a density of 4 × 10^5^ cells/well duplicate in 6-well plates and incubated with 1 mL of HDLDBC-bMPA(Rho(C17)_2_) at a final concentration of 10 μg of Rho(C17)_2_ at 37 °C. At different time-points between 15 min and 24 h, cells were washed twice with PBS, trypsinized and resuspended in 200 μL of PBS before analyzing events with a 523 nm laser in a FACSAria (Becton Dickinson).

### 4.6. Cell Viability Determination Based on Proliferation Assay and Flow Cytometry

To evaluate the cytotoxicity of the HDLDBCs, free and encapsulated CQ and CDDP, cells were seeded at a density of 5 × 10^3^ cells/well in 96-well plates and incubated for 24–72 h with 100 μL of HDLDBCs at 0.1–1 mg/mL, CQ between 1–100 μM and CDDP between 0.1–1 μg/mL final concentrations. AlamarBlue assay (Invitrogen) was used to evaluate cell proliferation following the standard protocol provided by the supplier. For the evaluation of the combination treatments, cells were seeded as before and treated with 100 μL of CQ at 40 μM or 80 μM. At 18 h, media were replaced with 100 μL of 0.5–1 μg/mL CDDP and cytotoxicity was evaluated after 48 h. The coefficient of drug interaction (CDI) was calculated as follows: CDI = AB/(A × B), where AB is the ratio of the viability of the combination groups to the control group, and A and B represent the ratio of viability of each drug. When CDI value < 1, the drugs are synergistic.

For cell cycle analysis, cells were seeded at a density of 5 × 10^3^ cells/well in 6-well plates and incubated with 40 μM of CQ. After 18 h, media were replaced with 1 μg/mL of CDDP for 48 h. Then, cells were trypsinized, washed twice with PBS, fixed on ice-cold 70% EtOH and maintained at 4 °C for at least 1 h. Samples were washed with cold PBS and stained with propidium iodide (PI) solution with RNase A (50 µg/mL PI, 0.1 mg/mL RNase A) before 106 events were measured using a FACSArray system (Becton Dickinson).

Apoptosis and necrosis were evaluated by double staining with Annexin V/propidium iodide (BD PharMingen). Cells were plated at 8 × 10^5^ cells into 60 mm-dishes, treated with 40 μM of CQ and/or 1 μg/mL of CDDP for 18–72 h, collected and washed with cold PBS. Cells were stained according to the manufacturer’s instructions and events were analyzed with a FACSAria (Becton Dickinson).

### 4.7. Statistical Analysis

Results are reported as mean ± SD, and experiments were performed at least in triplicate. Normally distributed data were analyzed by two-sample t test and one-way ANOVA using Minitab 17 software (State College, PA). *p* < 0.05 (*) and *p* < 0.01 (**) were considered to be statistically significant.

## Figures and Tables

**Table 1:** TEM, cryoTEM and DLS size data in nm measured for HDLDBC-bMPA and HDLDBC-bGMPA.

**HDLDBC-bMPA**			
**TEM**	**cryoTEM**	**DLS**	
		**Intensity**	**Number**
13 ± 3 22 ± 4	60 and 150	28 ± 4 (7%) 223 ± 80 (90%)	21 ± 6 (57%) 125 ± 54 (33%)
**HDLDBC-bGMPA**			
**TEM**	**cryoTEM**	**DLS**	
		**Intensity**	**Number**
13 ± 3	40–70	225 ± 89 (96%)	88 ± 49 (100%)

**Table 2:** Encapsulation efficiency of CQ and CDDP in HDLDBC-bMPA and HDLDBC-bGMPA nanocarriers. Hydrodynamic diameter of the aggregates with encapsulated drugs measured by DLS and given as intensity average.

**HDLDBC-bMPA**			
**Drug**	**EE (%) ^1^**	**mg drug/mg HDLDBC**	**Hydrodynamic Diameter (nm)**
**CQ**	49 ± 4%	0.49 ± 0.04	45 ± 8 (12%) and 200 ± 49 (88%)
**CDDP**	13 ± 11%	0.043 ± 0.014	18 ± 3 nm (3%) and 276 ± 76 nm (91%)
**HDLDBC-bGMPA**			
**Drug**	**EE (%) ^1^**	**mg drug/mg HDLDBC**	**Hydrodynamic Diameter (nm)**
**CQ**	67 ± 6%	0.67 ± 0.06	48 ± 18 nm (14%) and 318 ± 22 nm (86%)
**CDDP**	7%	0.017	49 ± 6 nm (8%) and 371 ± 20 nm (92%)

^1^ Encapsulation efficiency (EE,%) calculated as (weight of drug in the HDLDBC-bGMPA micelles/weight of the feeding drug) × 100%.

**Table 3:** Cytotoxic concentration values (CC50) for CQ and CDDP free or encapsulated on HDLDBCs against HeLa and A549 cell lines at different time points.

Cell Line	Time	CQ (µM)	HDLDBC-bMPA(CQ) (µM)	HDLDBC-bGMPA(CQ) (µM)	CDDP (µg/mL)	HDLDBC-bMPA(CDDP) (µg/mL)
HeLa	24 h	88.5	>100	>100	3.7	>5
	48 h	16.4	57.9	53.7	2.1	3.9
	72 h	7.8	19.5	33.9	2.3	3.5
A549	24 h	60.3	>100	>100	>5	>5
	48 h	44.7	>100	>100	3.3	>5
	72 h	41.0	70.0	100	2.6	>5

## Data Availability

Not applicable.

## Supplementary Materials

The following are available online at https://www.mdpi.com/article/10.3390/ijms22105223/s1. Figure S1: internalization curve of HDLDBC-bGMPA(Rho(C17)_2_) in human fibroblasts (Fdh) as analyzed by flow cytometry, Figure S2: release profile of Rho(C17)_2_ from HDLDBC-bMPA at 37 °C, Figure S3: TEM images of the encapsulation of CQ and CDDP in HDLDBC-bMPA and HDLDBC-bGMPA, Figure S4: release profile of CQ from HDLDBC-bMPA at 37 °C, Figure S5: cell viability of HeLa and A549 cells measured after 72 h of a simultaneous treatment with CQ and CDDP either free or encapsulated in HDLDBC-bMPA, Figure S6: alterations to the cell cycle and induction of apoptosis determined by flow cytometry after pre-treatment of HeLa and A549 cells for 18 h with 40 µM of free CQ or CQ encapsulated in HDLDBC-bMPA.
