# Supplementary material for: Combination Chemotherapy with Cisplatin and Chloroquine: Effect of Encapsulation in Micelles Formed by Self-Assembling Hybrid Dendritic–Linear–Dendritic Block Copolymers
Source: Int J Mol Sci. 2021 May 14;22(10):5223. doi: 10.3390/ijms22105223 (PMC8156097; doi:10.3390/ijms22105223)
Supplement: Supplementary file 1 [file ijms-22-05223-s001.zip › ijms-1199448-supplementary.pdf]

Combination Chemotherapy with Cisplatin and Chloroquine: Effect of Encapsulation in Micelles formed by Self-Assembling Hybrid Dendritic–Linear–Dendritic Block Copolymers

Preparation of double encapsulation of CQ and CDDP

HDLDBC-bMPA, CQ and CDDP were dissolved at the same time in a mixture of dichloromethane and distilled water (1:1)) at a feeding ratio of (1:1) (w_{HDLDBC}:w_{drug}) for CQ and at a feeding ratio of (1:0.25) (w_{HDLDBC}:w_{drug}) for CDDP. The samples were vigorously stirred at room temperature employing an orbital shaker under ventilation until complete evaporation of DCM (around 2 h). The non-encapsulated drugs were removed by dialysis at 4°C (regenerated cellulose membrane, Slide-A-Lyzer devices, MW 1000 Da cut-off, Thermo Scientific) against distilled water (200 mL) during 16 h. The amounts of encapsulated drugs were indirectly determined as described in Manuscript (*2.4 Drug encapsulation in HDLDBC nanoaggregates and releasing profiles*). 0.63 ± 0.09 mg of CQ per mg of HDLDBC-bMPA and 0.064 ± 0.052 mg of CDDP per mg of HDLDBC-bMPA were simultaneously encapsulated according to this protocol, giving encapsulation efficiency of 63 ± 9 % and 14 ± 4 % respectively. According to DLS, aggregates showing three size-populations were obtained: 27 ± 3 nm (4 %), 160 ± 21 nm (18 %) and 640 ± 109 nm (79 %).

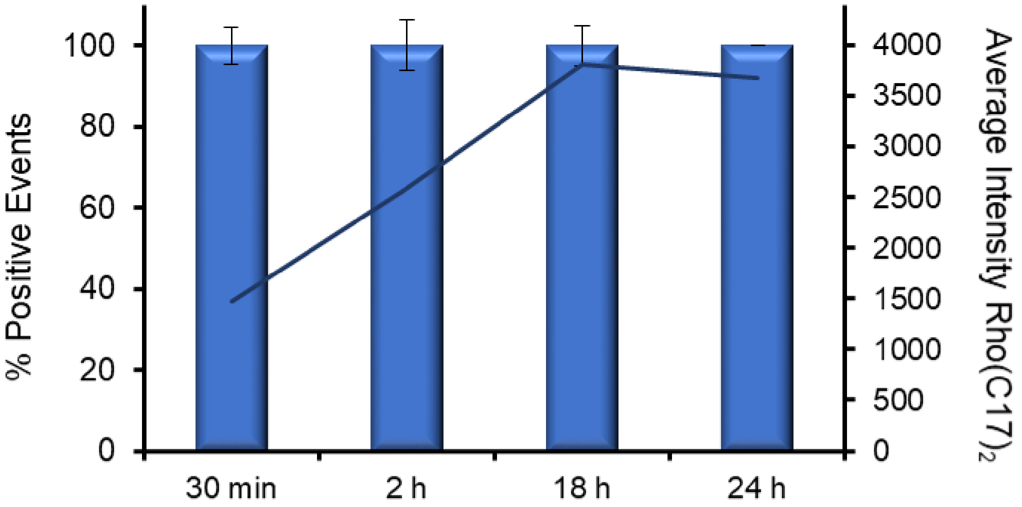

Figure S1. Internalization curve of HDLDBC-bGMPA(Rho(C17)$_2$) in human fibroblasts (Fdh) as analyzed by flow cytometry. Bars represent the percentage of rhodamine-positive events relative to the total number of events recorded. The line represents the average rhodamine intensity of the population at each time-point.

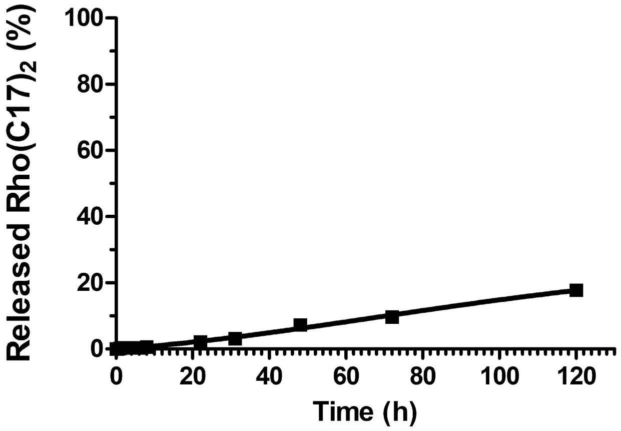

Figure S2. Release profile of $Rho(C17)_2$ from HDLDBC-bMPA at 37 °C. Data is expressed as percentage of total $Rho(C17)_2$ encapsulated at 0 h.

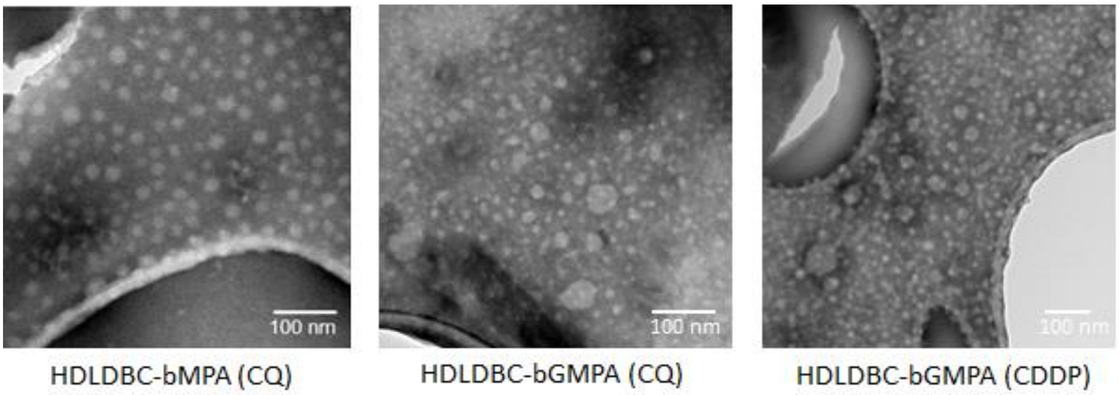

Figure S3. TEM images of the encapsulation of CQ and CDDPin HDLDBC-bMPA and HDLDBC-bGMPA.

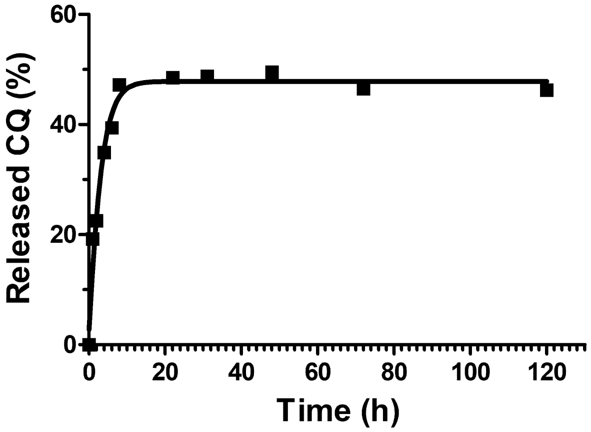

Figure S4. Release profile of CQ from HDLDBC-bMPA at 37 °C. Data is expressed as percentage of total CQ encapsulated at 0 h.

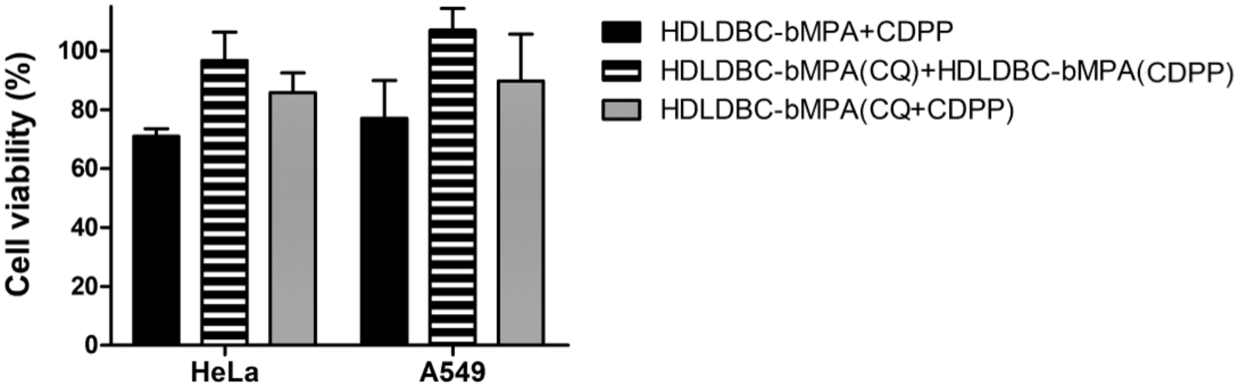

Figure S5. Cell viability of HeLa and A549 cells measured after 72 h of a simultaneous treatment with CQ and CDDP either free or encapsulated in HDLDBC-bMPA. Final concentrations used were as follows: HDLDBC-bMPA at 1 mg/mL and free CDDP at 1 μg/mL; HDLDBC-bMPA(CQ) at 40 μM of CQ and HDLDBC-bMPA(CDDP) at 1 μg/mL of CDDP; HDLDBC-bMPA(CQ + CDDP) at 40 μM of CQ and 4.4 μg/mL of CDDP.

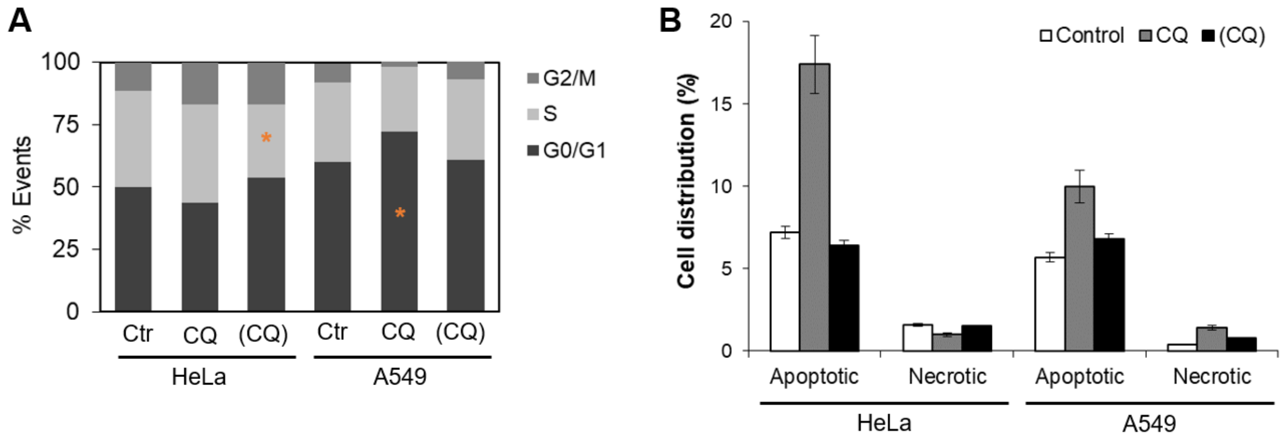

Figure S6. (**A**) Alterations to the cell cycle and (**B**) induction of apoptosis determined by flow cytometry after pre-treatment of HeLa and A549 cells for 18 h with 40 μM of free CQ or CQ encapsulated in HDLDBC-bMPA [(CQ)] (* indicates alterations >10% compared to control group).
